# *catena*-Poly[[(2,2′-bi­pyridine-κ^2^ *N*,*N*′)manganese(II)]-di-μ-bromido]

**DOI:** 10.1107/S2414314621000833

**Published:** 2021-01-29

**Authors:** Kwang Ha

**Affiliations:** a Chonnam National University, School of Chemical Engineering, Research Institute of Catalysis, Gwangju, Republic of Korea; Sunway University, Malaysia

**Keywords:** crystal structure, manganese(II) complex, 2,2′-bi­pyridine, polymeric complex.

## Abstract

In the polymeric title complex, [MnBr_2_(C_10_H_8_N_2_)]_*n*_, the Mn^II^ ion, situated on a twofold axis of symmetry, is six-coordinated in a distorted octa­hedral coordination geometry defined by two N atoms from the chelating 2,2′-bi­pyridine ligand and four bridging Br^−^ anions. The crystal reveals a one-dimensional Br-bridged chain along the *c-*axis direction with a zigzag topology. In the crystals, contacts between chains include π–π inter­actions between pyridyl rings [inter-centroid separation = 4.082 (1) Å]

## Abstract 2

The central Mn^II^ ion has a *cis*-N_2_Br_4_ octa­hedral coordination sphere defined by two N atoms of the bidentate 2,2′-bi­pyridine ligand and four bridging Br^−^ anions.

## Graphical abstract

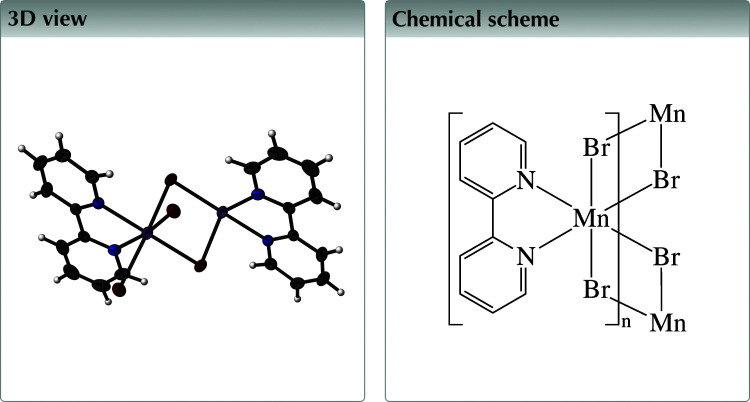

## Structure description

With reference to the title complex, [MnBr_2_(bipy)]_*n*_ (bipy = 2,2′-bi­pyridine), the crystal structures of related Mn^II^ complexes, namely [MnCl_2_(bipy)]_*n*_ (Lubben *et al.*, 1995 ▸) and [MnBr_2_(bipy)_2_] (Hwang & Ha, 2007 ▸) have been determined previously.

**Figure 1:**
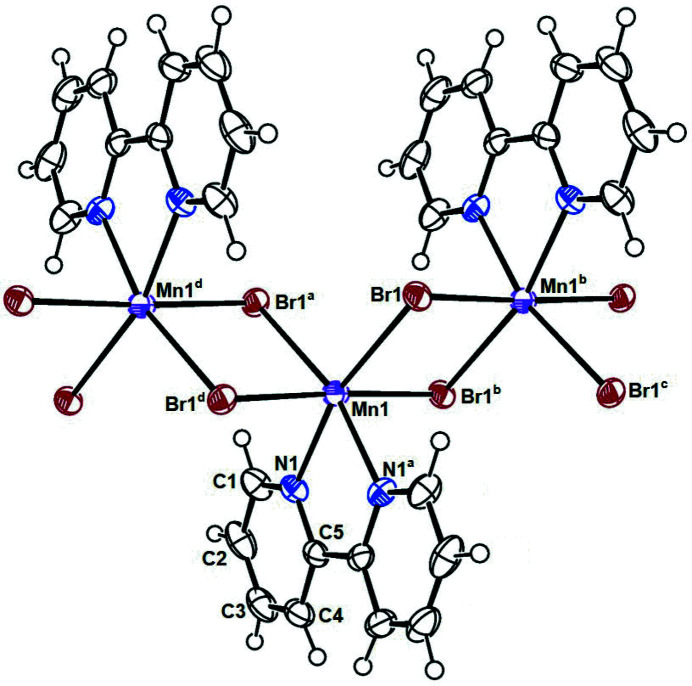
Part of the coordination polymer formed by the title complex showing the atom labelling and displacement ellipsoids drawn at the 50% probability level for non-H atoms. Symmetry codes: (*a*) −*x*, *y*, −*z* + 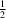 ; (*b*) −*x*, −*y*, −*z*; (*c*) *x*, −*y*, *z* − 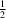 ; (*d*) *x*, −*y*, *z* + 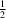 .

**Figure 2:**
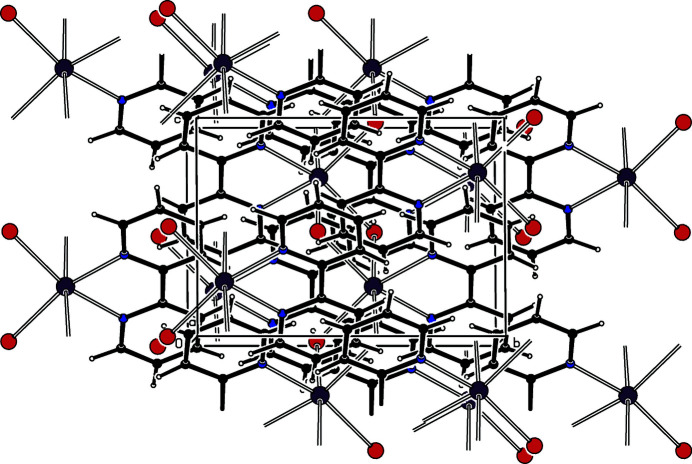
The packing in the crystal of the title complex, viewed approximately along the *a* axis.

In the title complex, the central Mn^II^ cation is six-coordinated within a distorted octa­hedral coordination geometry defined by two N atoms from chelating bipy ligand and four bridging Br^−^ anions (Fig. 1 ▸). The maximum deviation from the ideal octa­hedral angles is seen in the N1—Mn—N1^i^ chelate angle of 73.08 (7)°; symmetry operation (i): −*x*, *y*, −*z* + 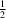 . The Mn ions are bridged by four bromido ligands to form a zigzag chain (glide symmetry) structure along the *c-*axis direction so the asymmetric unit of the polymer contains one half of the repeat unit, *i.e*. MnBr_2_(bipy); the Mn^II^ cation is situated on a twofold axis of symmetry. The Mn—Br bond lengths are somewhat different: the Mn—Br(*trans* to Br) distance of 2.7975 (2) Å is longer than the Mn—Br(*trans* to N) distance of 2.6373 (3) Å. The distance between adjacent Mn atoms is relatively short with the separation being 3.9656 (3) Å. The complex mol­ecules are stacked in columns along the *a* axis (Fig. 2 ▸). In the columns, several inter­molecular π–π inter­actions between adjacent pyridine rings are present. The closest contact involves *Cg*1 (the centroid of ring N1,C1–C5) and *Cg*1^ii^ [symmetry code: (ii) *x*, −*y* + 1, *z* + 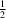 ], the centroid–centroid distance is 4.082 (1) Å and the dihedral angle between the ring planes is 8.79 (9)°.

## Synthesis and crystallization

To a solution of [MnBr_2_(bipy)_2_] (0.2713 g, 0.515 mmol) in 2-meth­oxy­ethanol (30 ml) was added MnBr_2_·4H_2_O (0.1491 g, 0.520 mmol), followed by reflux for 2 h. After cooling, the formed precipitate was separated by filtration, washed with ethanol and ether, and dried at 323 K, to give a pale-yellow powder (0.2671 g). Pale-yellow crystals of the product suitable for X-ray analysis were obtained by slow evaporation from its 2-meth­oxy­ethanol solution at room temperature.

## Refinement

Crystal data, data collection and structure refinement details are summarized in Table 1 ▸.

## Supplementary Material

Crystal structure: contains datablock(s) I. DOI: 10.1107/S2414314621000833/tk4066sup1.cif

Structure factors: contains datablock(s) I. DOI: 10.1107/S2414314621000833/tk4066Isup2.hkl

CCDC reference: 2058386

Additional supporting information:  crystallographic information; 3D view; checkCIF report

## Figures and Tables

**Table 1:** Experimental details

Crystal data	
Chemical formula	[MnBr_2_(C_10_H_8_N_2_)]
*M*_r_	370.94
Crystal system, space group	Monoclinic, *C*2/*c*
Temperature (K)	223
*a*, *b*, *c* (Å)	17.3039 (9), 9.5255 (5), 7.1852 (3)
β (°)	109.0347 (15)
*V* (Å^3^)	1119.57 (10)
*Z*	4
Radiation type	Mo *K*α
μ (mm^−1^)	8.28
Crystal size (mm)	0.29 × 0.14 × 0.05

Data collection	
Diffractometer	PHOTON 100 CMOS detector
Absorption correction	Multi-scan (*SADABS*; Bruker, 2016 ▸)
*T*_min_, *T*_max_	0.373, 0.745
No. of measured, independent and observed [*I* > 2σ(*I*)] reflections	14090, 1068, 1037
*R*_int_	0.038
(sin θ/λ)_max_ (Å^−1^)	0.618

Refinement	
*R*[*F* ^2^ > 2σ(*F* ^2^)], *wR*(*F* ^2^), *S*	0.013, 0.034, 1.11
No. of reflections	1068
No. of parameters	85
H-atom treatment	All H-atom parameters refined
Δρ_max_, Δρ_min_ (e Å^−3^)	0.23, −0.23

Computer programs: *APEX2* and *SAINT* (Bruker, 2016 ▸), *SHELXT2014/7* (Sheldrick, 2015*a* ▸), *SHELXL2014/7* (Sheldrick, 2015*b* ▸) and *ORTEP-3 for Windows* (Farrugia, 2012 ▸).

## full crystallographic data

### Crystal data

**Table:**

[MnBr_2_(C_10_H_8_N_2_)]	*F*(000) = 708
*M**_r_* = 370.94	*D*_x_ = 2.201 Mg m^−^^3^
Monoclinic, *C*2/*c*	Mo *K*α radiation, λ = 0.71073 Å
*a* = 17.3039 (9) Å	Cell parameters from 9930 reflections
*b* = 9.5255 (5) Å	θ = 3.6–26.0°
*c* = 7.1852 (3) Å	µ = 8.28 mm^−^^1^
β = 109.0347 (15)°	*T* = 223 K
*V* = 1119.57 (10) Å^3^	Block, pale yellow
*Z* = 4	0.29 × 0.14 × 0.05 mm

### Data collection

**Table:**

PHOTON 100 CMOS detector diffractometer	1037 reflections with *I* > 2σ(*I*)
Radiation source: sealed tube	*R*_int_ = 0.038
φ and ω scans	θ_max_ = 26.0°, θ_min_ = 3.6°
Absorption correction: multi-scan (SADABS; Bruker, 2016)	*h* = −21→21
*T*_min_ = 0.373, *T*_max_ = 0.745	*k* = −11→11
14090 measured reflections	*l* = −8→8
1068 independent reflections	

### Refinement

**Table:**

Refinement on *F*^2^	Primary atom site location: structure-invariant direct methods
Least-squares matrix: full	Secondary atom site location: difference Fourier map
*R*[*F*^2^ > 2σ(*F*^2^)] = 0.013	Hydrogen site location: difference Fourier map
*wR*(*F*^2^) = 0.034	All H-atom parameters refined
*S* = 1.11	*w* = 1/[σ^2^(*F*_o_^2^) + (0.0095*P*)^2^ + 1.0722*P*] where *P* = (*F*_o_^2^ + 2*F*_c_^2^)/3
1068 reflections	(Δ/σ)_max_ < 0.001
85 parameters	Δρ_max_ = 0.23 e Å^−^^3^
0 restraints	Δρ_min_ = −0.22 e Å^−^^3^

### Special details

**Table:**

Geometry. All esds (except the esd in the dihedral angle between two l.s. planes) are estimated using the full covariance matrix. The cell esds are taken into account individually in the estimation of esds in distances, angles and torsion angles; correlations between esds in cell parameters are only used when they are defined by crystal symmetry. An approximate (isotropic) treatment of cell esds is used for estimating esds involving l.s. planes.
Refinement. All H atoms were located from Fourier difference maps and refined isotropically; C—H = 0.93 (2)–0.97 (2) Å.

### Fractional atomic coordinates and isotropic or equivalent isotropic displacement parameters (Å^2^)

**Table:**

	*x*	*y*	*z*	*U*_iso_*/*U*_eq_	
Mn1	0.0000	0.08813 (3)	0.2500	0.02456 (9)	
Br1	−0.09377 (2)	−0.09461 (2)	0.00183 (2)	0.02780 (8)	
N1	0.07232 (8)	0.27734 (14)	0.39308 (19)	0.0259 (3)	
C1	0.14754 (10)	0.2716 (2)	0.5270 (3)	0.0339 (4)	
C2	0.19474 (12)	0.3903 (2)	0.5944 (3)	0.0411 (4)	
C3	0.16249 (12)	0.5191 (2)	0.5240 (3)	0.0416 (4)	
C4	0.08538 (12)	0.52703 (19)	0.3900 (3)	0.0354 (4)	
C5	0.04134 (10)	0.40425 (15)	0.3247 (2)	0.0251 (3)	
H1	0.1647 (13)	0.178 (3)	0.573 (3)	0.046 (6)*	
H2	0.2483 (15)	0.379 (2)	0.692 (3)	0.043 (6)*	
H3	0.1942 (17)	0.599 (2)	0.561 (4)	0.053 (7)*	
H4	0.0607 (15)	0.612 (2)	0.333 (4)	0.049 (6)*	

### Atomic displacement parameters (Å^2^)

**Table:**

	*U*^11^	*U*^22^	*U*^33^	*U*^12^	*U*^13^	*U*^23^
Mn1	0.02373 (17)	0.02100 (17)	0.02314 (17)	0.000	−0.00031 (13)	0.000
Br1	0.02714 (10)	0.02824 (11)	0.02510 (10)	−0.00752 (6)	0.00451 (7)	−0.00407 (5)
N1	0.0229 (6)	0.0269 (7)	0.0271 (6)	−0.0011 (5)	0.0070 (5)	−0.0057 (5)
C1	0.0246 (8)	0.0390 (10)	0.0346 (9)	0.0008 (7)	0.0047 (7)	−0.0101 (7)
C2	0.0284 (9)	0.0578 (12)	0.0360 (10)	−0.0106 (8)	0.0091 (8)	−0.0203 (8)
C3	0.0470 (10)	0.0427 (11)	0.0395 (10)	−0.0210 (9)	0.0203 (8)	−0.0179 (8)
C4	0.0487 (10)	0.0276 (9)	0.0365 (9)	−0.0090 (8)	0.0227 (8)	−0.0072 (7)
C5	0.0299 (8)	0.0251 (8)	0.0262 (8)	−0.0030 (6)	0.0173 (7)	−0.0040 (6)

### Geometric parameters (Å, º)

**Table:**

Mn1—N1^i^	2.2433 (13)	C1—C2	1.386 (3)
Mn1—N1	2.2433 (13)	C1—H1	0.97 (2)
Mn1—Br1^i^	2.6373 (3)	C2—C3	1.375 (3)
Mn1—Br1	2.6373 (3)	C2—H2	0.97 (2)
Mn1—Br1^ii^	2.7975 (2)	C3—C4	1.369 (3)
Mn1—Br1^iii^	2.7975 (2)	C3—H3	0.93 (2)
Br1—Mn1^ii^	2.7975 (2)	C4—C5	1.391 (2)
N1—C1	1.344 (2)	C4—H4	0.95 (2)
N1—C5	1.348 (2)	C5—C5^i^	1.482 (3)
			
N1^i^—Mn1—N1	73.08 (7)	C1—N1—Mn1	124.10 (11)
N1^i^—Mn1—Br1^i^	165.56 (4)	C5—N1—Mn1	117.21 (10)
N1—Mn1—Br1^i^	95.29 (3)	N1—C1—C2	122.67 (18)
N1^i^—Mn1—Br1	95.29 (3)	N1—C1—H1	113.6 (13)
N1—Mn1—Br1	165.56 (4)	C2—C1—H1	123.7 (13)
Br1^i^—Mn1—Br1	97.395 (13)	C3—C2—C1	118.50 (18)
N1^i^—Mn1—Br1^ii^	92.32 (3)	C3—C2—H2	122.7 (13)
N1—Mn1—Br1^ii^	85.65 (3)	C1—C2—H2	118.8 (13)
Br1^i^—Mn1—Br1^ii^	95.344 (7)	C4—C3—C2	119.58 (17)
Br1—Mn1—Br1^ii^	86.331 (6)	C4—C3—H3	120.3 (16)
N1^i^—Mn1—Br1^iii^	85.65 (3)	C2—C3—H3	120.0 (16)
N1—Mn1—Br1^iii^	92.31 (3)	C3—C4—C5	119.46 (18)
Br1^i^—Mn1—Br1^iii^	86.331 (6)	C3—C4—H4	123.3 (15)
Br1—Mn1—Br1^iii^	95.344 (7)	C5—C4—H4	117.1 (15)
Br1^ii^—Mn1—Br1^iii^	177.470 (13)	N1—C5—C4	121.44 (16)
Mn1—Br1—Mn1^ii^	93.669 (6)	N1—C5—C5^i^	116.04 (9)
C1—N1—C5	118.32 (14)	C4—C5—C5^i^	122.51 (11)
			
C5—N1—C1—C2	−1.2 (2)	Mn1—N1—C5—C4	−173.49 (11)
Mn1—N1—C1—C2	171.71 (13)	C1—N1—C5—C5^i^	179.20 (16)
N1—C1—C2—C3	1.3 (3)	Mn1—N1—C5—C5^i^	5.8 (2)
C1—C2—C3—C4	−0.1 (3)	C3—C4—C5—N1	1.2 (2)
C2—C3—C4—C5	−1.1 (3)	C3—C4—C5—C5^i^	−178.03 (18)
C1—N1—C5—C4	−0.1 (2)		

Symmetry codes: (i) −*x*, *y*, −*z*+1/2; (ii) −*x*, −*y*, −*z*; (iii) *x*, −*y*, *z*+1/2.
